# Supplementary material for: Modification Effects of Population Expansion, Ageing, and Adaptation on Heat-Related Mortality Risks Under Different Climate Change Scenarios in Guangzhou, China
Source: Int J Environ Res Public Health. 2019 Jan 29;16(3):376. doi: 10.3390/ijerph16030376 (PMC6388188; doi:10.3390/ijerph16030376)
Supplement: Supplementary file 1 [file ijerph-16-00376-s001.pdf]

International Journal of
Environmental Research and Public Health

Supplemental Material

Modification effects of urban expansion, ageing and adaptation on heat-related mortality risks under different climate change scenarios

Tao Liu, Zhoupeng Ren, Yonghui Zhang, Baixiang Feng, Hualiang Lin, Jianpeng Xiao, Weilin Zeng, Xing Li, Zhihao Li, Shannon Rutherford, Yanjun Xu, Shao Lin, Philip C Nasca, Yaodong Du, Jinfeng Wang, Cunrui Huang, Qingfeng Du, Peng Jia, Wenjun Ma

International Journal of
Environmental Research and Public Health
MDPI

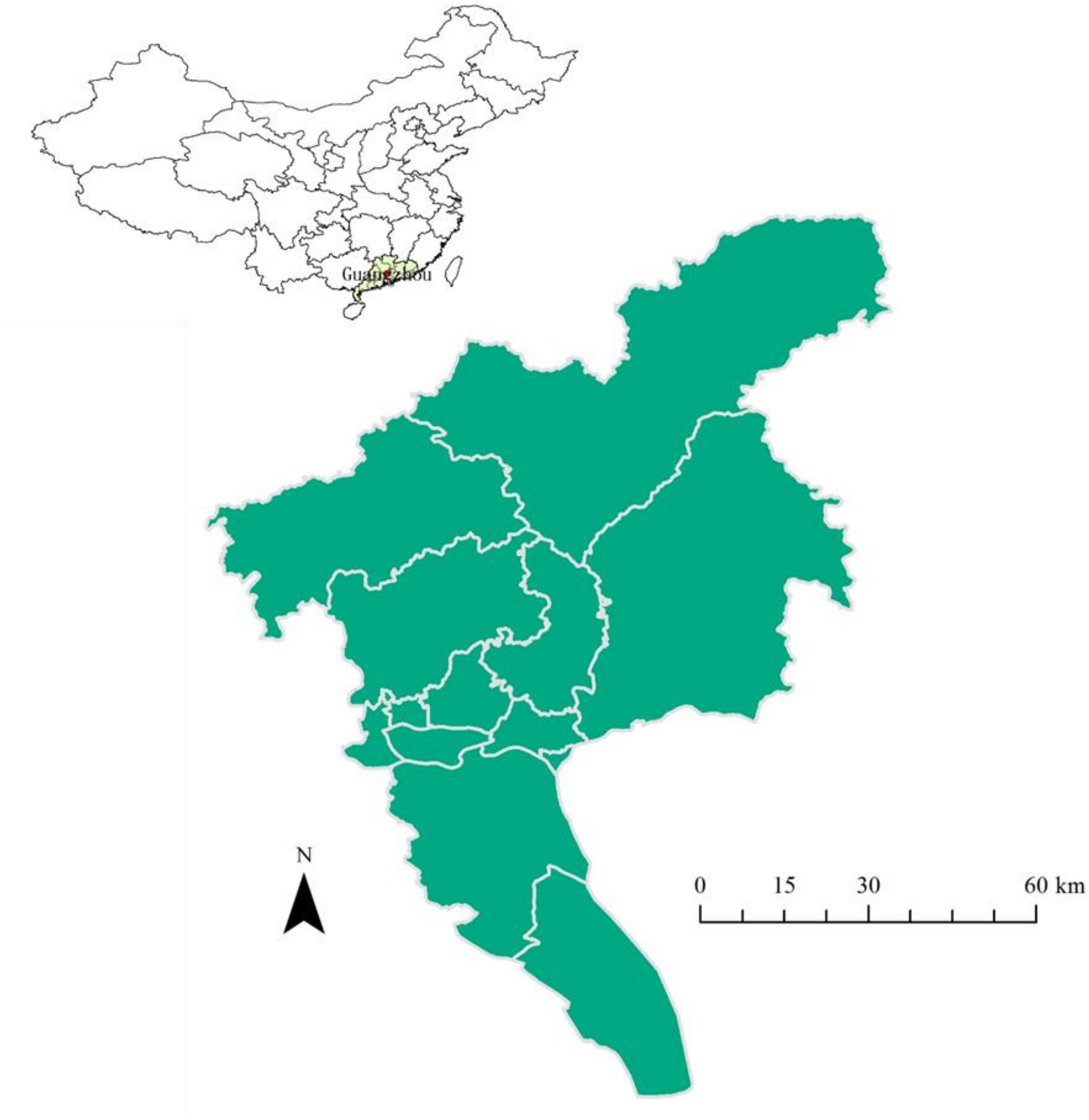

Figure S1. The location of Guangzhou, China.

International Journal of
Environmental Research and Public Health

MDPI

Table S1. Detailed information of 27 CMIP5 GCM models.

Model name	Modelling center	Horizontal resolution (lat×lon, degree)	RCP2·6	RCP4·5	RCP8·5
**ACCESS1-0**	Commonwealth Scientific and Industrial Research Organization (CSIRO) and Bureau of Meteorology (BOM), Australia	1·25×1·875		√	√
**ACCESS1-3**		1·25×1·875		√	√
**bcc_csm1_1**	Beijing Climate Center, China Meteorological Administration	2·77×2·81	√	√	√
**bcc_csm1_1_m**		1·1×1·1	√	√	√
**BNU-ESM**	College of Global Change and Earth System Science, Beijing Normal University	2·8×2·8	√	√	√
**CanESM2**	Canadian Centre for Climate Modelling and Analysis	2·8×2·8	√	√	√
**CCSM4**	National Center for Atmospheric Research	0·9×1·25	√	√	√
**CESM1-BGC**	National Science Foundation, Department of Energy, National Center for Atmospheric Research	0·9×1·25		√	√
**CESM1-CAM5**		0·9×1·25	√	√	√
**CMCC-CM**	Centro Euro-Mediterraneo per I Cambiamenti Climatici	0·75×0·75		√	√
**CMCC-CMS**		1·86×1·875		√	√
**CNRM-CM5**	Centre National de Recherches Meteorologiques, Meteo-France, France	1·40×1·41	√	√	√
**CSIRO-Mk3-6-0**	Commonwealth Scientific and Industrial Research Organization in collaboration with the Queensland Climate Change Centre of Excellence	1·86×1·875	√	√	√
**GFDL-CM3**	NOAA Geophysical Fluid Dynamics Laboratory, USA	2·0×2·5	√	√	√
**GFDL-ESM2G**		2·0×2·5	√	√	√
**GFDL-ESM2M**		2·0×2·5		√	√
**Inmcm4**	Institute for Numerical Mathematics, Russia	1·5×2·0		√	√
**IPSL-CM5A-LR**	Institute Pierre-Simon Laplace, France	1·875×3·75	√	√	√
**IPSL-CM5A-MR**		1·25×2·5	√	√	
**IPSL-CM5B-LR**		1·875×3·75			√
**MIROC5**	Atmosphere and Ocean Research Institute (The University of Tokyo), National Institute for Environmental Studies, and Japan Agency for Marine-Earth Science and Technology	1·39×1·41	√	√	√
**MIROC-ESM**	Japan Agency for Marine-Earth Science and Technology, Atmosphere and Ocean Research Institute (The University of Tokyo), and National Institute for Environmental Studies	1·77×2·81	√	√	√
**MIROC-ESM-CHEM**		1·77×2·81	√	√	√
**MPI-ESM-LR**	Max Planck Institute for Meteorology, Germany	1·85×1·875	√	√	√
**MPI-ESM-MR**		1·85×1·875	√		√
**MRI-CGCM3**	Meteorological Research Institute, Japan	1·125×1·125	√	√	√
**NorESM1-M**	Norwegian Climate Centre	1·875×2·5	√	√	√

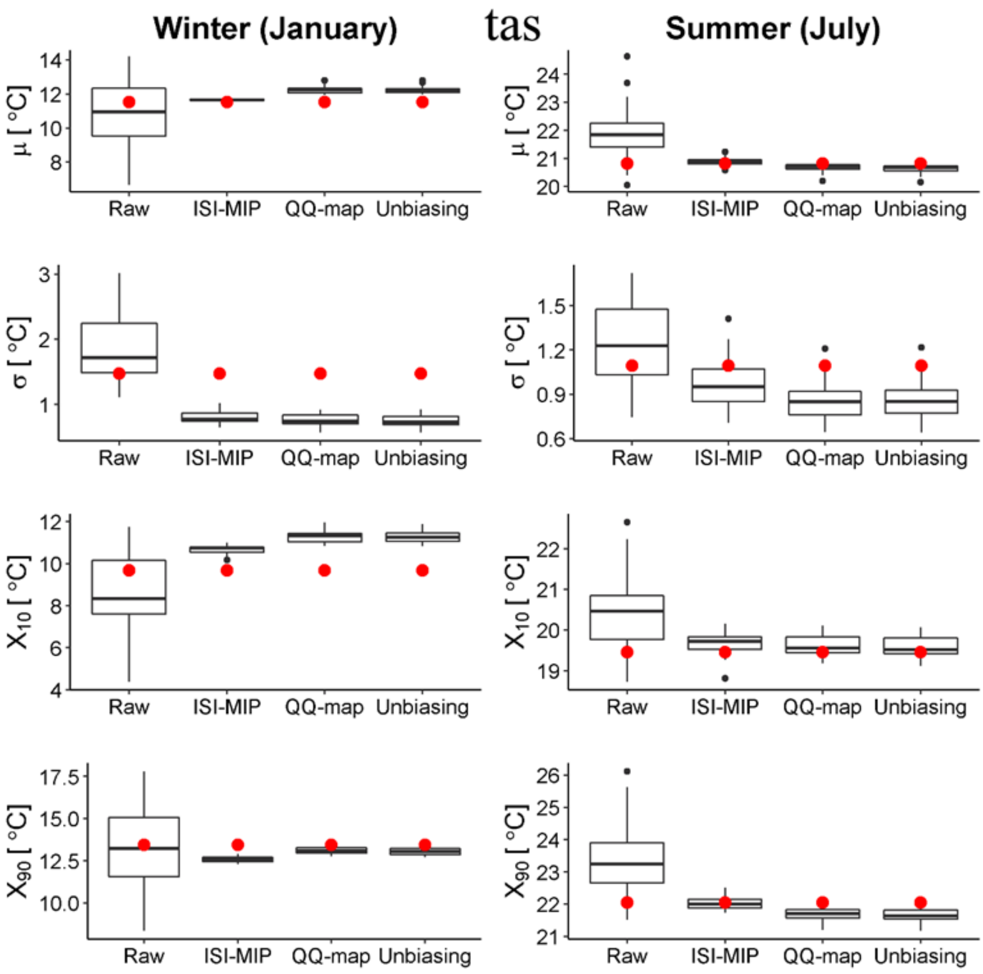

Figure S2. Comparison of different bias correction methods adjusting the GCM outputs.

All analyses were conducted using 27 GCM outputs and daily observed TM for 680 stations in China from 1960 to 1999. We assessed the difference between daily observed TM (red dot) and adjusted GCM outputs (box plot) corrected by different methods during winter and summer. Four indexes were employed: mean (μ), standard deviation (σ), 10th and 90th percentiles (X_{10}, X_{90}).

International Journal of
Environmental Research and Public Health
MDPI

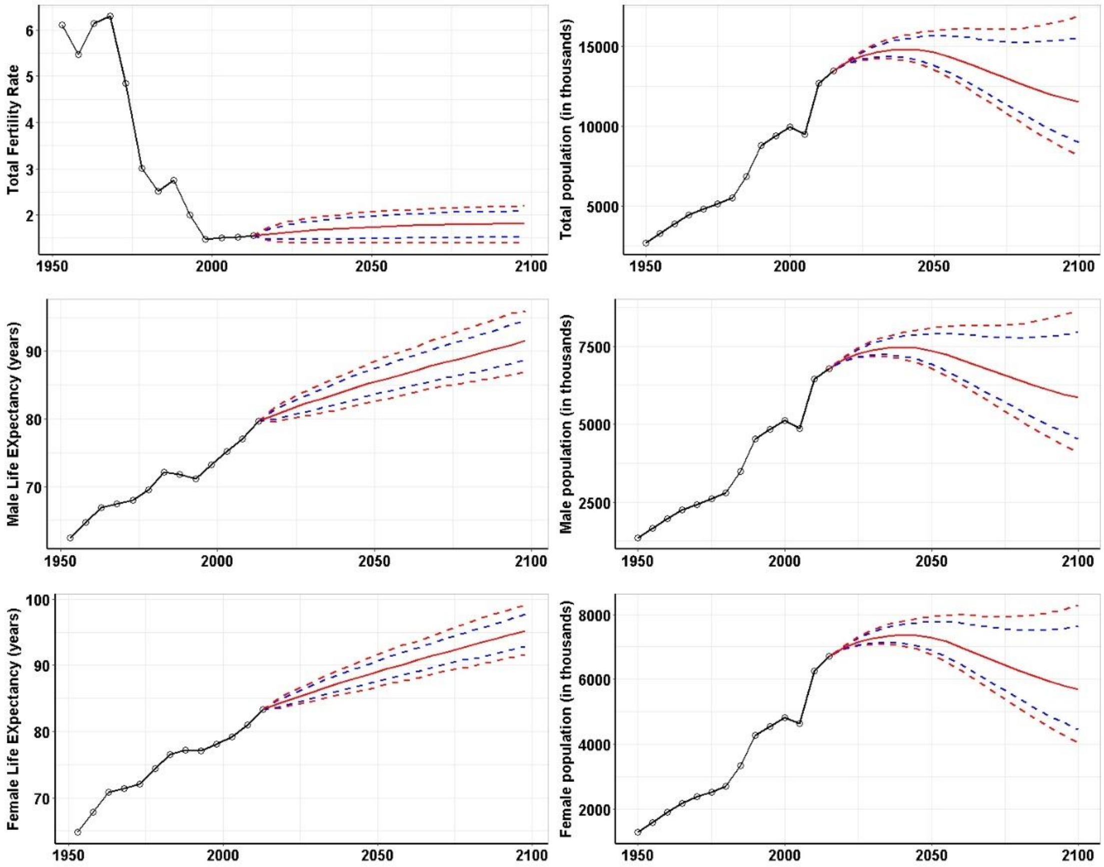

Figure S3. Bayesian probabilistic population projections for Guangzhou, 2020–2100: major population indicators.

The historical observed data are shown by a black line. The Bayesian predictive distributions are shown in red: median—solid; 80% prediction interval— blue dashed; 90% prediction interval— red dashed.

International Journal of
Environmental Research and Public Health

Table S2. Population projections in Guangzhou in the 2030s, 2060s and 2090s under different scenarios.

	Population increase scenario		
	**Low**	**Medium**	**High**
**2030s**			
**Total population (*1000)**	14,229	14,773	15,518
**Male population (*1000)**	7,148	7,436	7,832
**Female population (*1000)**	7,082	7,337	7,686
**Population<65 years (*1000)**	11,012	11,266	11,691
**Population≥65 years (*1000)**	3,217	3,507	3,827
**Percentage of elderly population (%)**	22·61	23·74	24·66
**2060s**			
**Total population (*1000)**	11,974	13,710	16,103
**Male population (*1000)**	5,987	6,895	8,146
**Female population (*1000)**	5,988	6,815	7,956
**Population<65 years (*1000)**	8,312	8,817	9,493
**Population≥65 years (*1000)**	3,662	4,893	6,610
**Percentage of elderly population (%)**	30·58	35·69	41·05
**2090s**			
**Total population (*1000)**	8,601	11,761	16,655
**Male population (*1000)**	4,321	5,961	8,500
**Female population (*1000)**	4,280	5,800	8,156
**Population<65 years (*1000)**	6,088	7,533	9,040
**Population≥65 years (*1000)**	2,513	4,228	7,615
**Percentage of elderly population (%)**	29·22	35·95	45·72

Low: The 10th percentile of future 10,000 projection trajectories. Medium: The median value of future 10,000 projection trajectories. High: The 90th percentile of future 10,000 projection trajectories. Elderly: Population≥65 years.

International Journal of *Environmental Research and Public Health*

MDPI

Table S3. Annual temperature (℃) in the 2030s, 2060s and 2090s under different climatic scenarios and models.

Climatic Model	Mean (SD) temperature								
	RCP 2·6			RCP 4·5			RCP8·5		
	2030s	2060s	2090s	2030s	2060s	2090s	2030s	2060s	2090s
ACCESS1-0	-	-	-	21·9(6·6)	22·6(6·2)	23·1(6·0)	21·9(6·7)	23·4(5·9)	24·6(5·0)
ACCESS1-3	-	-	-	21·4(6·8)	22·3(6·4)	22·4(6·2)	21·6(6·7)	22·8(5·8)	23·7(5·1)
bcc-csm1-1	21·9(7·0)	21·9(7·1)	21·7(7·2)	21·8(7·0)	22·2(7·0)	22·5(6·7)	22·0(7·0)	22·8(6·6)	23·8(5·9)
bcc-csm1-1-m	21·8(6·9)	21·9(6·8)	21·6(7·1)	21·8(6·9)	22·1(6·9)	22·2(6·9)	21·8(7·0)	22·6(6·6)	23·4(6·1)
BNU-ESM	21·4(7·4)	21·6(7·4)	21·6(7·3)	21·6(7·4)	22·3(6·9)	22·4(7·0)	21·9(7·2)	22·8(6·7)	23·6(6·1)
CanESM2	22·3(7·0)	22·2(7·1)	22·0(7·2)	22·1(7·1)	22·5(7·1)	22·9(6·8)	22·4(7·0)	23·0(6·6)	23·4(5·8)
CCSM4	21·5(7·2)	21·5(7·1)	21·5(7·1)	21·6(7·1)	22·0(6·9)	22·0(6·9)	21·5(7·2)	22·5(6·7)	23·5(6·0)
CESM1-BGC		-	-	21·5(7·1)	21·9(7·0)	22·2(6·7)	21·6(7·1)	22·5(6·6)	23·1(6·2)
CESM1-CAM5	21·8(6·9)	22·1(6·8)	22·3(6·8)	21·7(7·0)	22·5(6·8)	23·1(6·6)	22·0(6·9)	2·3·4(6·3)	24·4(5·8)
CMCC-CM	-	-	-	21·8(7·0)	22·6(6·5)	22·9(6·4)	22·1(6·8)	23·1(6·2)	24·2(5·4)
CMCC-CMS	-	-	-	22·0(6·8)	22·7(6·3)	23·2(6·0)	22·0(6·7)	23·4(5·9)	24·7(4·9)
CNRM-CM5	21·5(7·3)	21·7(7·3)	21·6(7·2)	21·5(7·4)	22·1(7·0)	22·3(7·0)	21·7(7·2)	22·6(6·9)	-
CSIRO-Mk3-6-0	21·8(6·6)	22·1(6·4)	22·1(6·5)	21·8(6·6)	22·5(6·3)	22·8(5·9)	21·8(6·8)	23·0(5·8)	23·9(4·8)
GFDL-CM3	22·1(7·0)	22·5(6·6)	22·4(6·6)	22·1(6·9)	22·5(6·6)	22·9(6·2)	22·1(6·9)	23·0(6·2)	23·5(5·3)
GFDL-ESM2G	21·3(7·6)	21·1(7·7)	21·1(7·6)	21·2(7·4)	21·4(7·4)	21·6(7·4)	21·3(7·5)	22·2(7·0)	22·4(6·8)
GFDL-ESM2M	-	-	-	21·5(7·8)	22·0(7·6)	21·9(7·8)	21·8(7·6)	22·3(7·4)	23·0(7·0)
Inmcm4	-	-	-	21·2(7·8)	21·5(7·7)	21·6(7·7)	21·4(7·7)	22·2(7·5)	23·0(7·1)
IPSL-CM5A-LR	21·8(6·8)	22·1(6·6)	21·9(6·9)	22·2(6·5)	22·6(6·5)	22·8(6·4)	22·2(6·5)	23·4(5·9)	24·1(5·3)
IPSL-CM5A-MR	21·6(7·1)	22·0(6·9)	21·8(7·0)	22·0(6·9)	22·3(6·9)	22·8(6·5)	22·0(7·0)	23·2(6·4)	24·4(5·4)
IPSL-CM5B-LR	-	-	-	21·6(7·1)	22·1(6·8)	22·3(6·7)	21·8(7·0)	22·9(6·3)	23·4(5·9)
MIROC-ESM-CHEM	21·7(7·9)	22·0(7·8)	21·9(7·8)	21·7(7·9)	22·4(7·7)	22·6(7·5)	21·9(7·9)	22·8(·7·4)	24·4(6·6)
MIROC-ESM	22·0(7·7)	22·0(7·5)	22·3(7·6)	22·0(7·6)	22·6(7·5)	22·8(7·4)	21·8(7·7)	22·9(7·4)	24·1(6·8)
MIROC5	21·6(6·8)	22·1(6·5)	22·1(6·5)	21·8(6·6)	22·6(6·2)	22·6(6·2)	21·9(6·7)	22·9(5·9)	23·7(5·1)
MPI-ESM-LR	21·8(6·8)	21·7(6·9)	21·7(6·8)	22·1(6·7)	22·4(6·5)	22·5(6·4)	22·1(6·6)	23·2(6·0)	24·2(5·4)
MPI-ESM-MR	21·7(7·1)	21·9(6·9)	21·8(7·0)	21·9(6·9)	22·4(6·7)	22·5(6·5)	22·0(6·8)	23·1(6·1)	24·1(5·3)
MRI-CGCM3	21·3(7·0)	21·6(6·9)	21·8(6·7)	21·6(6·8)	21·9(6·6)	22·2(6·5)	21·5(6·8)	22·4(6·3)	23·4(5·6)
NorESM1-M	21·4(7·3)	21·8(7·0)	21·8(7·1)	21·8(7·1)	22·2(6·9)	22·4(6·9)	21·8(7·1)	23·0(6·5)	24·1(5·9)
Average	21·7	21·9	21·8	21·8	22·3	22·5	21·9	22·9	23·8

-: Data not available.

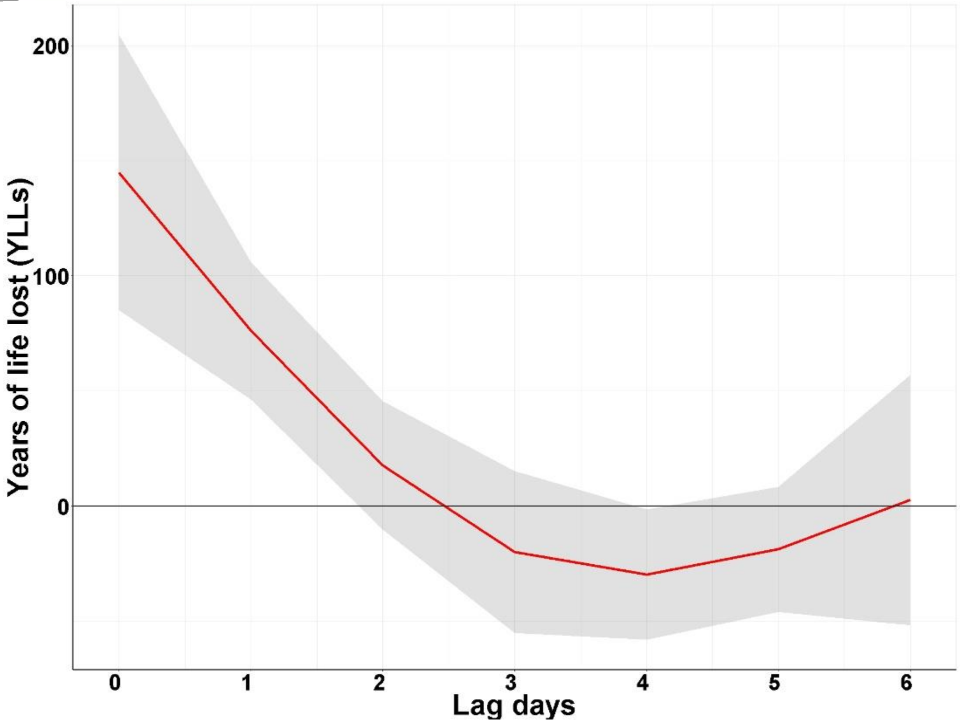

Figure S4. Lag effects of TM (30℃) on YLLs in the total population during 2010-2015 in Guangzhou, China.

Note: All effects of TM on YLLs were adjusted for secular trend, wind speed, day of week, relative humidity, SO_2, NO_2 and PM_{10}. The reference temperature was 23·0℃ in the DLNM model. Lag effect: The heat effects mainly appeared during the fist two days.

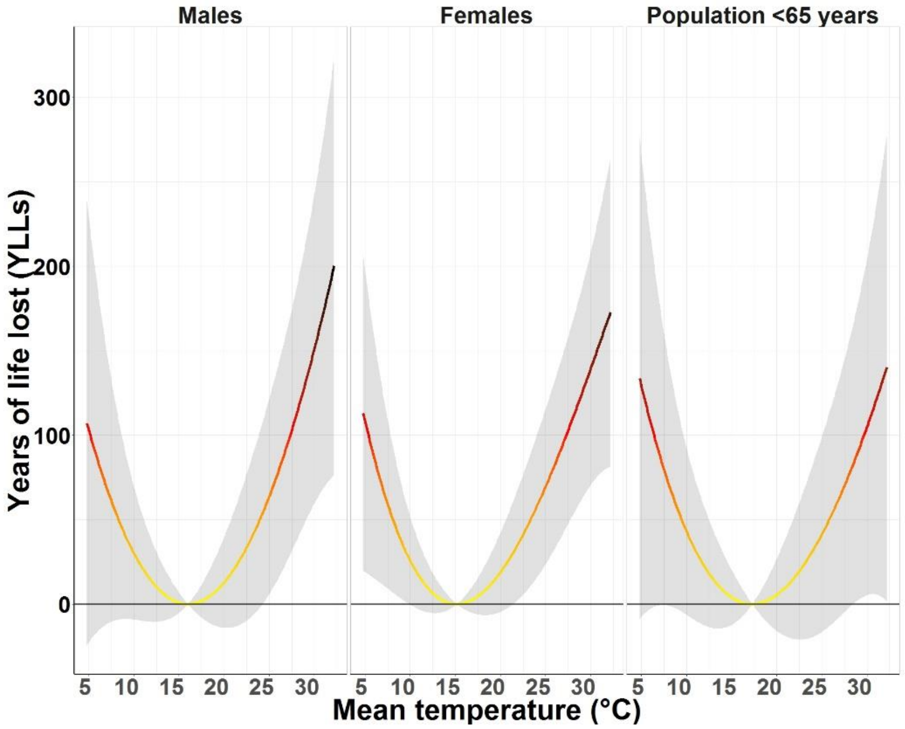

Figure S5. Relationship between daily TM and YLLs in males, females and the population < 65 years during 2010-2015 in Guangzhou, China.

Note: All effects of TM on YLLs were adjusted for secular trend, wind speed, day of week, relative humidity, SO_2, NO_2 and PM_{10}.

International Journal of *Environmental Research and Public Health*

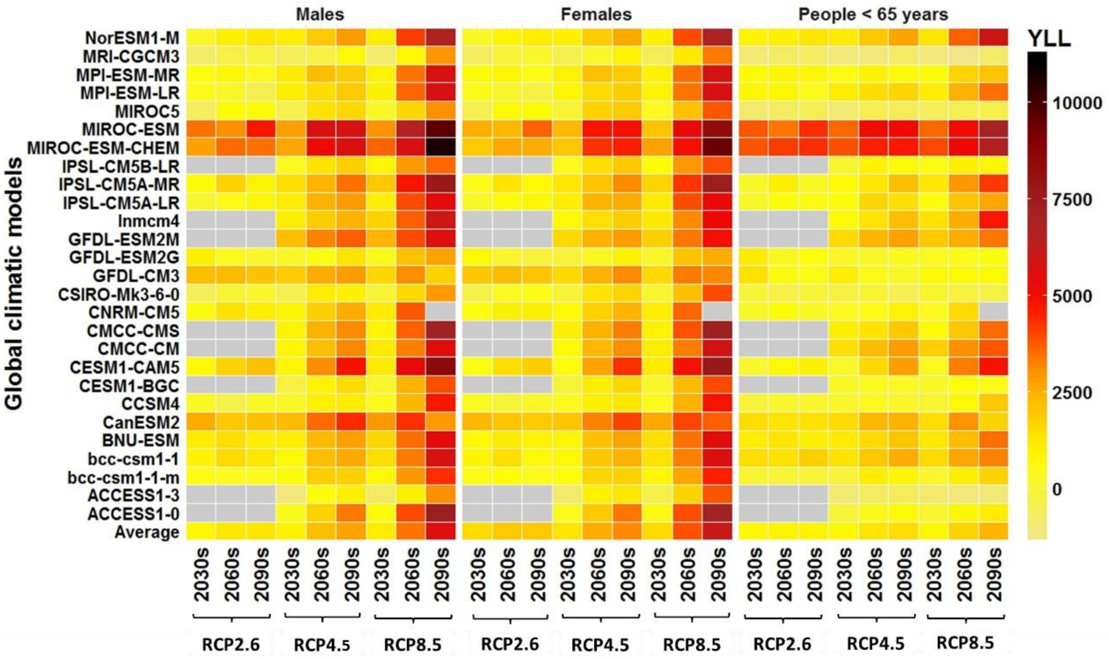

Figure S6. Annual heat-related YLLs in males, females and population <65 years in the 2030s, 2060s and 2090s as compared with the 1980s under different climatic scenarios and GCMs.

Note: We assumed that the population size and their adaptation in the 21st century will remain constant at the 2010 level. Gray grids meant the data were not available. The heat-related YLLs in the future have been subtracted by the heat-related YLLs in the 1980s.

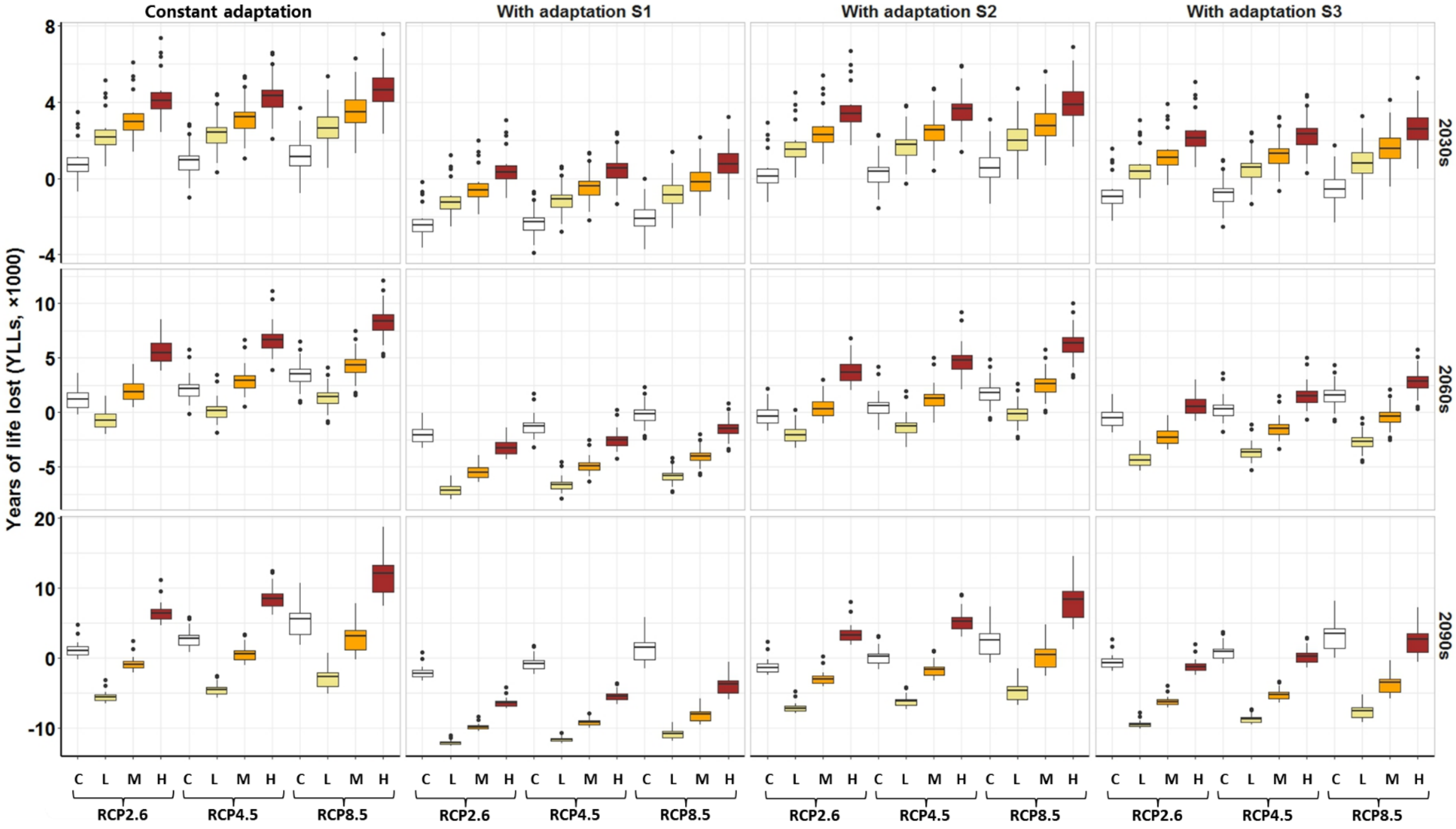

Figure S7. Impacts of population expansion and adaptation on the annual heat-related YLLs in males in Guangzhou in the 2030s, 2060s and 2090s under different scenarios.

International Journal of Environmental Research and Public Health

MDPI

Constant adaptation: People's adaptation to high temperature will remain constant at the 2010 level.
Adaptation S1: People's adaptation to high temperature will increase by 8·92% per decade.
Adaptation S2: People's adaptation to high temperature will increase by 4·60% per decade.
Adaptation S3: People's adaptation to high temperature will increase by 0·2℃ per decade.
C: The population size will remain constant at the 2010 level.
L: Low population expansion scenario.
M: Medium population expansion scenario.
H: High population expansion scenario.
The heat-related YLLs in the future have been subtracted by the heat-related YLLs in the 1980s.

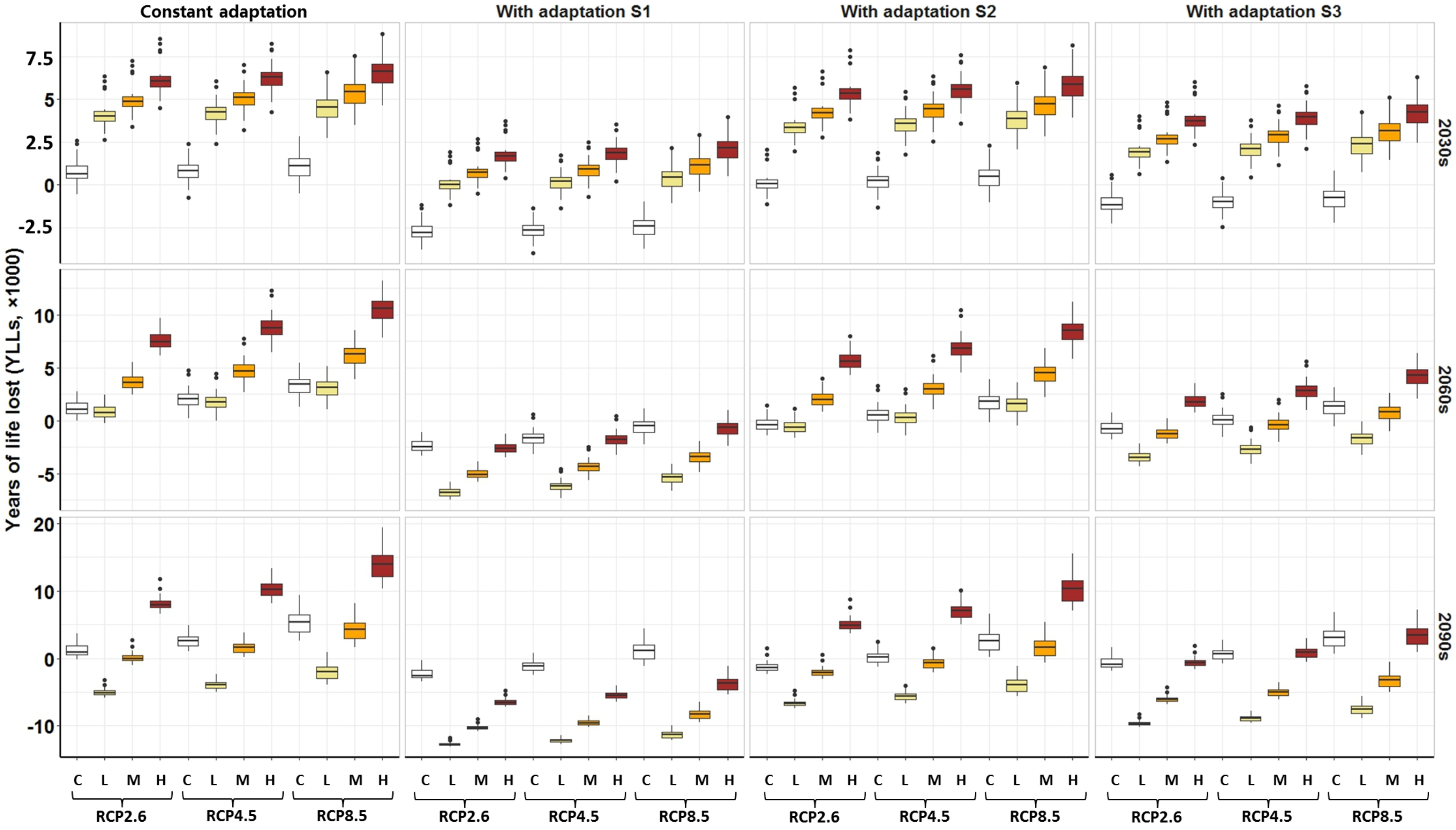

Figure S8. Impacts of population expansion and adaptation on the annual heat-related YLLs in females in Guangzhou in the 2030s, 2060s and 2090s under different scenarios.

International Journal of
Environmental Research and Public Health
MDPI

Constant adaptation: People's adaptation to high temperature will remain constant at the 2010 level.
Adaptation S1: People's adaptation to high temperature will increase by 8·92% per decade.
Adaptation S2: People's adaptation to high temperature will increase by 4·60% per decade.
Adaptation S3: People's adaptation to high temperature will increase by 0·2℃ per decade.
C: The population size will remain constant at the 2010 level.
L: Low population expansion scenario.
M: Medium population expansion scenario.
H: High population expansion scenario.
The heat-related YLLs in the future have been subtracted by the heat-related YLLs in the 1980s.

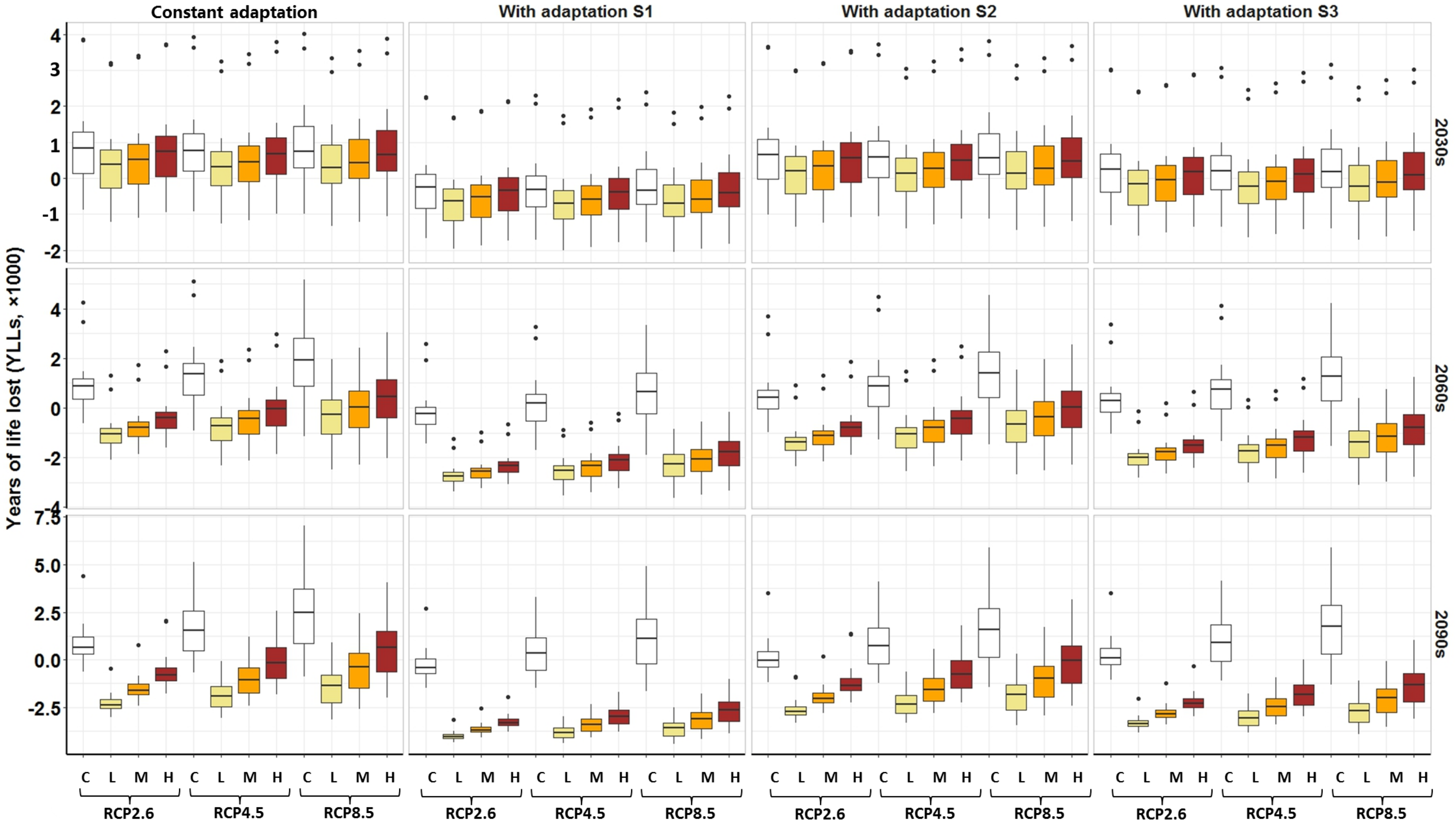

Figure S9. Impacts of population expansion and adaptation on the annual heat-related YLLs in people <65 years in Guangzhou in the 2030s, 2060s and 2090s under different scenarios.

International Journal of Environmental Research and Public Health

MDPI

Constant adaptation: People's adaptation to high temperature will remain constant at the 2010 level.

Adaptation S1: People's adaptation to high temperature will increase by 8·92% per decade.

Adaptation S2: People's adaptation to high temperature will increase by 4·60% per decade.

Adaptation S3: People's adaptation to high temperature will increase by 0·2℃ per decade.

C: The population size will remain constant at the 2010 level.

L: Low population expansion scenario.

M: Medium population expansion scenario.

H: High population expansion scenario.

The heat-related YLLs in the future have been subtracted by the heat-related YLLs in the 1980s.

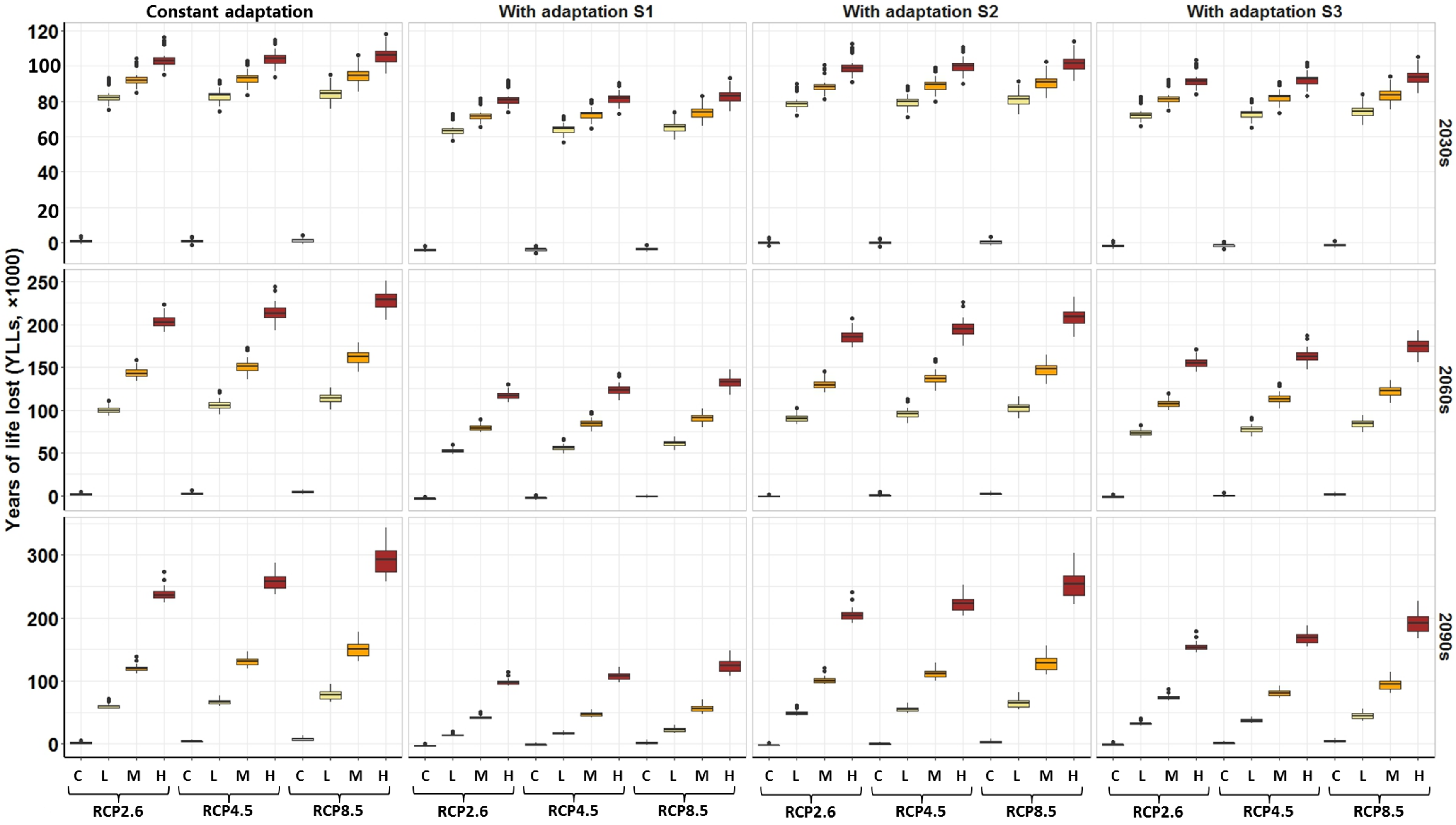

Figure S10. Impacts of population expansion and adaptation on the annual heat-related YLLs in the elderly population in Guangzhou in the 2030s, 2060s and 2090s under different scenarios.

International Journal of
Environmental Research and Public Health
MDPI

Constant adaptation: People's adaptation to high temperature will remain constant at the 2010 level.
Adaptation S1: People's adaptation to high temperature will increase by 8·92% per decade.
Adaptation S2: People's adaptation to high temperature will increase by 4·60% per decade.
Adaptation S3: People's adaptation to high temperature will increase by 0·2℃ per decade.
C: The population size will remain constant at the 2010 level.
L: Low population expansion scenario.
M: Medium population expansion scenario.
H: High population expansion scenario.
The heat-related YLLs in the future have been subtracted by the heat-related YLLs in the 1980s.

International Journal of *Environmental Research and Public Health*

MDPI

Table S4. Increase rate (β, P) of heat-related YLLs for each unit increase in population size and aging degree under different scenarios.

	Constant adaptation			With adaptation S1			With adaptation S2			With adaptation S3		
	**RCP2·6**	**RCP4·5**	**RCP8·5**	**RCP2·6**	**RCP4·5**	**RCP8·5**	**RCP2·6**	**RCP4·5**	**RCP8·5**	**RCP2·6**	**RCP4·5**	**RCP8·5**
**2030s**												
**Total population (×1000)***	4·23 (0·005)	4·23 (0·005)	4·29 (0·005)	3·51 (0·005)	3·51 (0·005)	3·56 (0·005)	4·10 (0·005)	4·10 (0·005)	4·15 (0·005)	3·85 (0·005)	3·85 (0·005)	3·90 (0·005)
**Males (×1000)***	1·89 (0·003)	1·90 (0·003)	1·92 (0·003)	1·57 (0·003)	1·57 (0·003)	1·60 (0·003)	1·83 (0·003)	1·84 (0·003)	1·86 (0·003)	1·72 (0·003)	1·72 (0·003)	1·75 (0·003)
**Females (×1000)***	2·03 (0·002)	2·03 (0·002)	2·06 (0·002)	1·68 (0·002)	1·68 (0·002)	1·71 (0·002)	1·97 (0·002)	1·97 (0·002)	2·00 (0·002)	1·85 (0·002)	1·85 (0·002)	1·87 (0·002)
**People <65 years (×1000)***	0·64 (0·004)	0·63 (0·004)	0·64 (0·004)	0·53 (0·004)	0·52 (0·004)	0·53 (0·004)	0·62 (0·004)	0·61 (0·004)	0·62 (0·004)	0·58 (0·004)	0·57 (0·004)	0·58 (0·004)
**Elderly(×1000)***	2·89 (<0·001)	2·90 (<0·001)	2·93 (<0·001)	2·40 (<0·001)	2·40 (<0·001)	2·43 (<0·001)	2·80 (<0·001)	2·81 (<0·001)	2·84 (<0·001)	2·63 (<0·001)	2·64 (<0·001)	2·67 (<0·001)
**Aging degree#**	423·3 (0·015)	423·3 (0·015)	428·0 (0·015)	364·6 (0·017)	364·8 (0·017)	368·5 (0·017)	412·5 (0·015)	412·8 (0·015)	417·1 (0·015)	392·4 (0·016)	392·6 (0·016)	396·6 (0·016)
**2060s**												
**Total population (×1000)***	4·28 (0·001)	4·48 (0·001)	4·75 (0·001)	2·68 (0·001)	2·81 (0·001)	2·98 (0·001)	3·94 (0·001)	4·12 (0v001)	4·37 (0·001)	3·38 (0·001)	3·54 (0.001)	3·75 (0·001)
**Males (×1000)***	3··10 (0·015)	3·26 (0·015)	3·46 (0·015)	1·94 (0v015)	2·04 (0·015)	2·17 (0·015)	2·86 (0·015)	3·00 (0·015)	3·19 (0·015)	2·45 (0·015)	2·57 (0·015)	2·73 (0·015)
**Females(×1000)***	3·56 (0·014)	3·72 (0·014)	3·94 (0·014)	2·23 (0·014)	2·33 (0·014)	2·47 (0·014)	3·31 (0·014)	3·47 (0·014)	3·67 (0·014)	2·81 (0·014)	2·94 (0·014)	3·11 (0·014)
**People <65 years (×1000)***	1·14 (0·047)	1·21 (0·047)	1·30 (0·047)	0··71 (0··047)	0·76 (0.047)	0·81 (0·047)	1·06 (0·047)	1·12 (0·047)	1·21 (0·047)	0·90 (0·047)	0·95 (0·047)	1·03 (0·047)
**Elderly(×1000)***	17·57 (0·014)	18·34 (0·014)	19·41 (0·014)	11·01 (0·014)	11·49 (0·014)	12·17 (0·014)	16·2 (0·014)	16·92 (0·014)	17·92 (0·014)	13·88 (0·014)	14·49 (0·014)	15·34 (0·014)
**Aging degree#**	407·3 (0·001)	422·7 (0·001)	444·1 (0·001)	276·5 (0·001)	286·1 (0·001)	299·6 (0·001)	380·0 (0·001)	394·3 (0·001)	4143 (0·001)	333·8 (0·001)	345·9 (0·001)	3629 (0·001)
**2090s**												
**Total population (×1000)***	4·28 (<0·001)	459 (<0·001)	5·17 (<0·001)	2·03 (<0·001)	2·17 (<0·001)	2·45 (<0·001)	3·74 (<0·001)	4·01 (<0·001)	4·52 (<0·001)	2·94 (<0·001)	3·15 (<0·001)	3·55 (<0·001)
**Males (×1000)***	3·79 (0·039)	4·08 (0·039)	4·60 (0·039)	1·80 (0·039)	1·93 (0·039)	2·18 (0·039)	3·32 (0·039)	3·58 (0·039)	4·03 (0·039)	260 (0·039)	2·80 (0·039)	3·16 (0·039)
**Females(×1000)***	4·28 (0·034)	4·59 (0·034)	5·17 (0·034)	2·03 (0·034)	2·17 (0·034)	245 (0·034)	3·81 (0·034)	409 (0·034)	4·61 (0·034)	2·94 (0·034)	3·15 (0·034)	3·55 (0·034)
**People <65 years (×1000)***	0·74 (0·048)	080 (0·048)	0·92 (0·048)	0·35 (0··048)	0·38 (0·048)	0·44 (0·048)	0·66 (0·048)	0·71 (0·048)	0·82 (0048)	0·51 (0·048)	0·55 (0·048)	0·63 (0048)
**Elderly(×1000)***	38·59 (0·033)	41·3 (0·033)	46·45 (0·033)	18·27 (0·033)	19·56 (0·033)	22·0 (0·033)	33·84 (0·033)	36·24 (0·033)	40·79 (0·033)	26·47 (0·033)	28·34 (0·033)	31·87 (0·033)
**Aging degree#**	443·1 (0·023)	467·7 (0·022)	514·4 (0··02)	258·9 (0·039)	270·5 (0·038)	292·6 (0·035)	400·1 (0·025)	421·8 (0·024)	463·1 (0·022)	333·3 (0·031)	350·1 (0·029)	382·2 (0·027)

Constant adaptation: People's adaptation to high temperature will remain constant at the 2010 level.

Adaptation S1: People's adaptation to high temperature will increase by 8·92% per decade.

Adaptation S2: People's adaptation to high temperature will increase by 4·60% per decade.

Adaptation S3: People's adaptation to high temperature will increase by 0·2℃ per decade.

β is the coefficient in the linear regression between heat-related YLLs and the percentage of population increase and aging degree.

The heat-related YLLs in the future have been subtracted by the heat-related YLLs in the 1980s.
